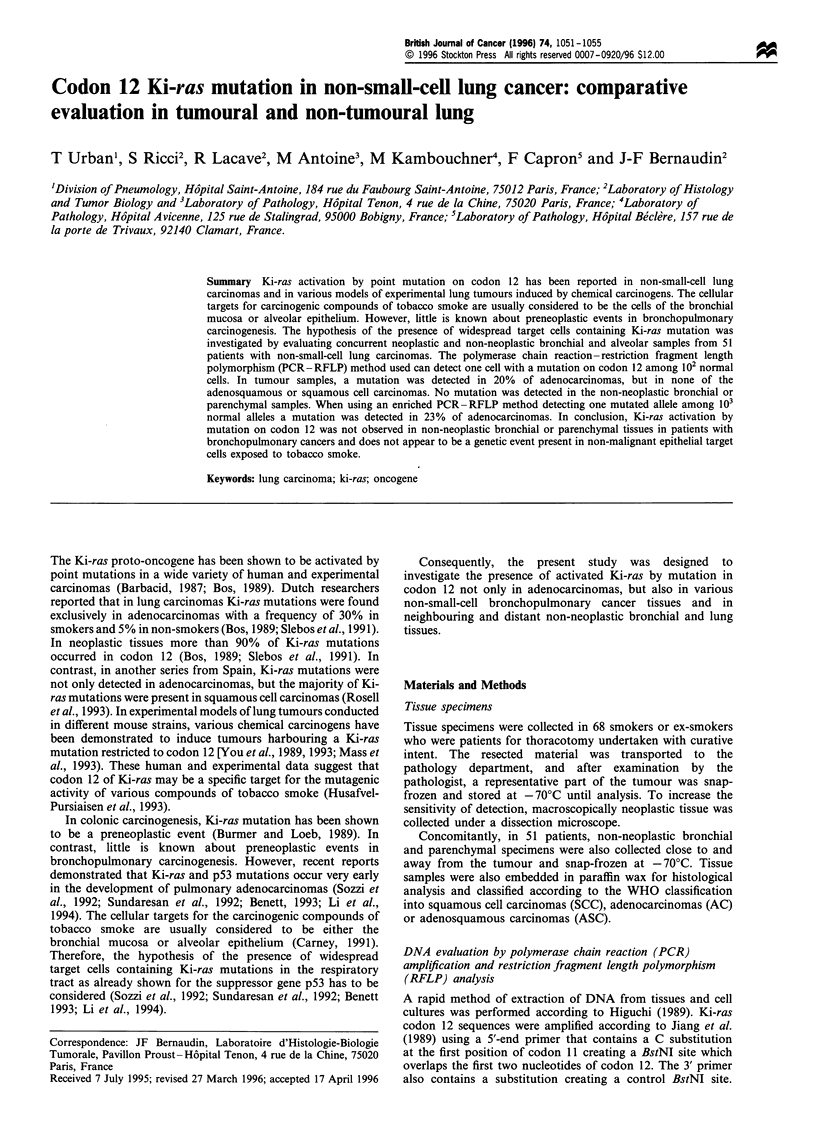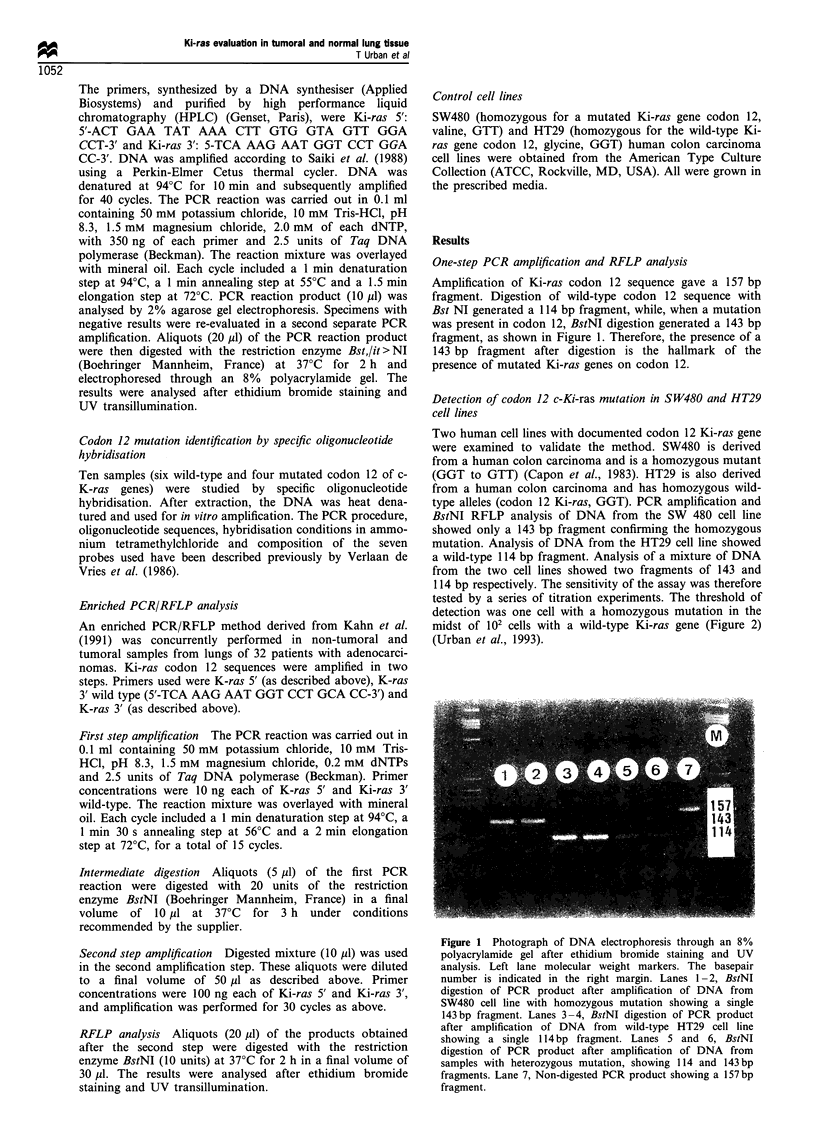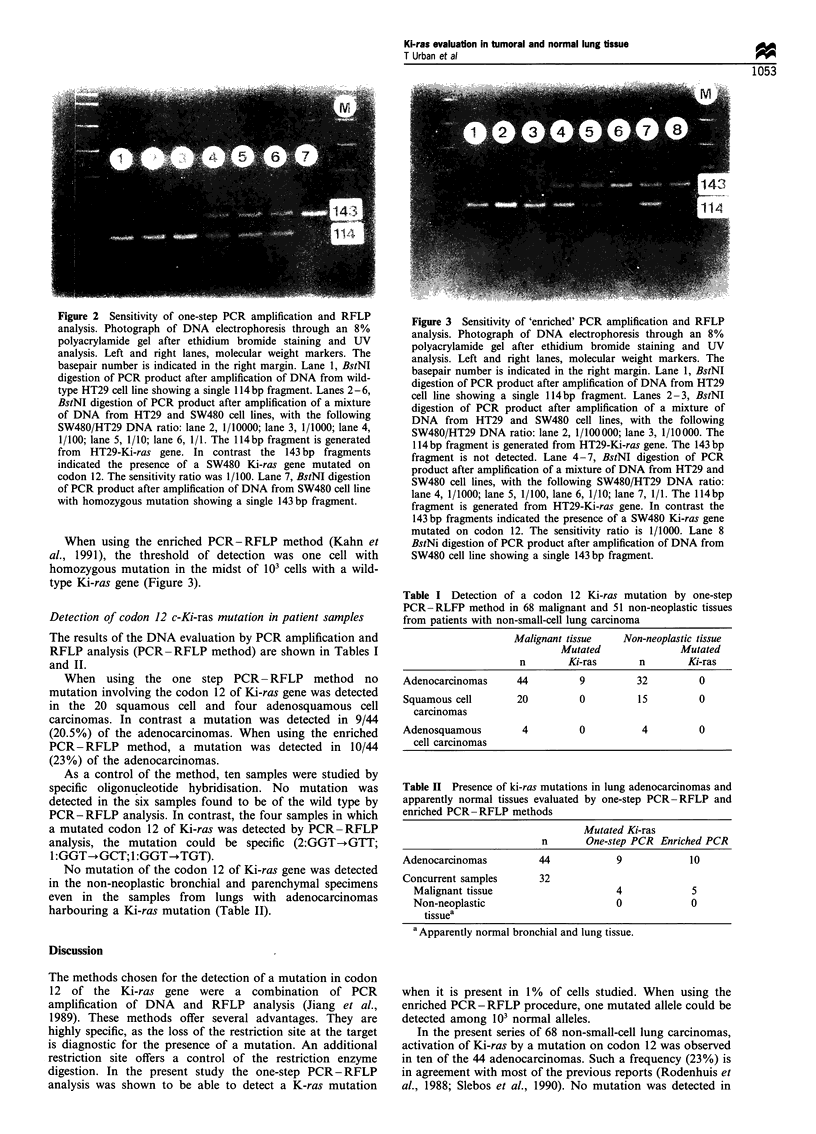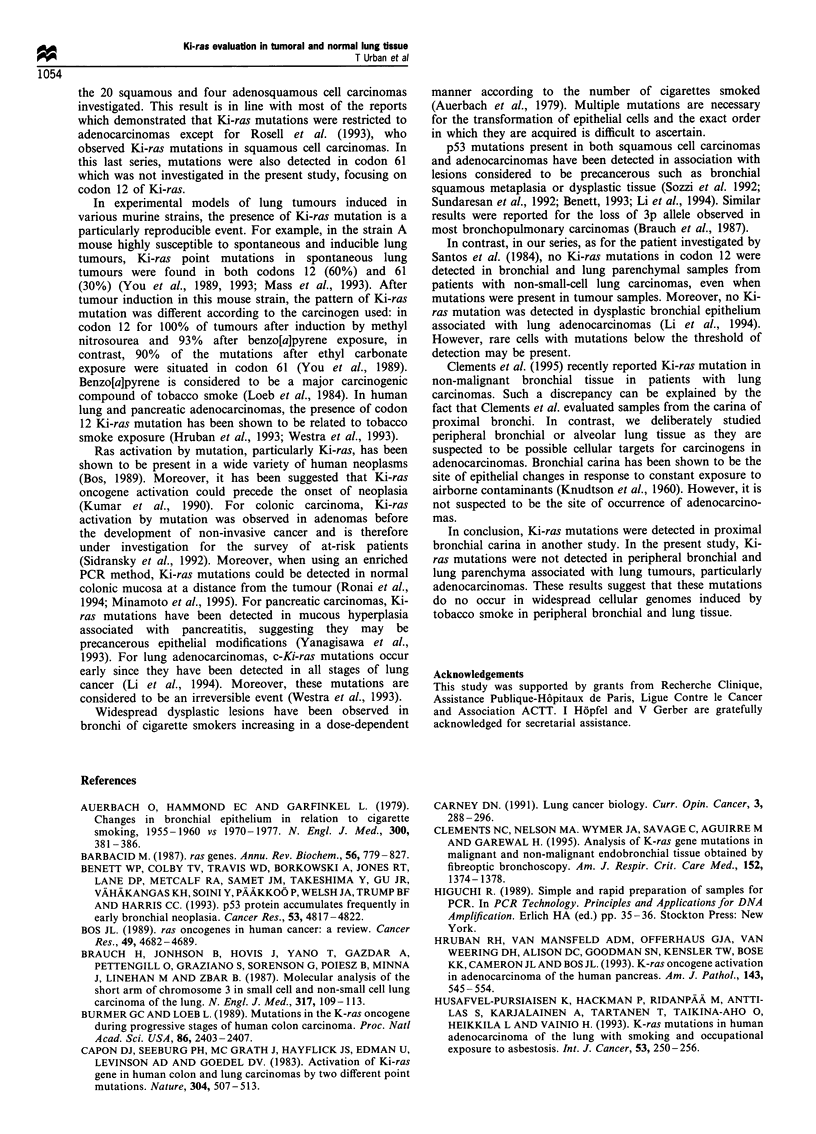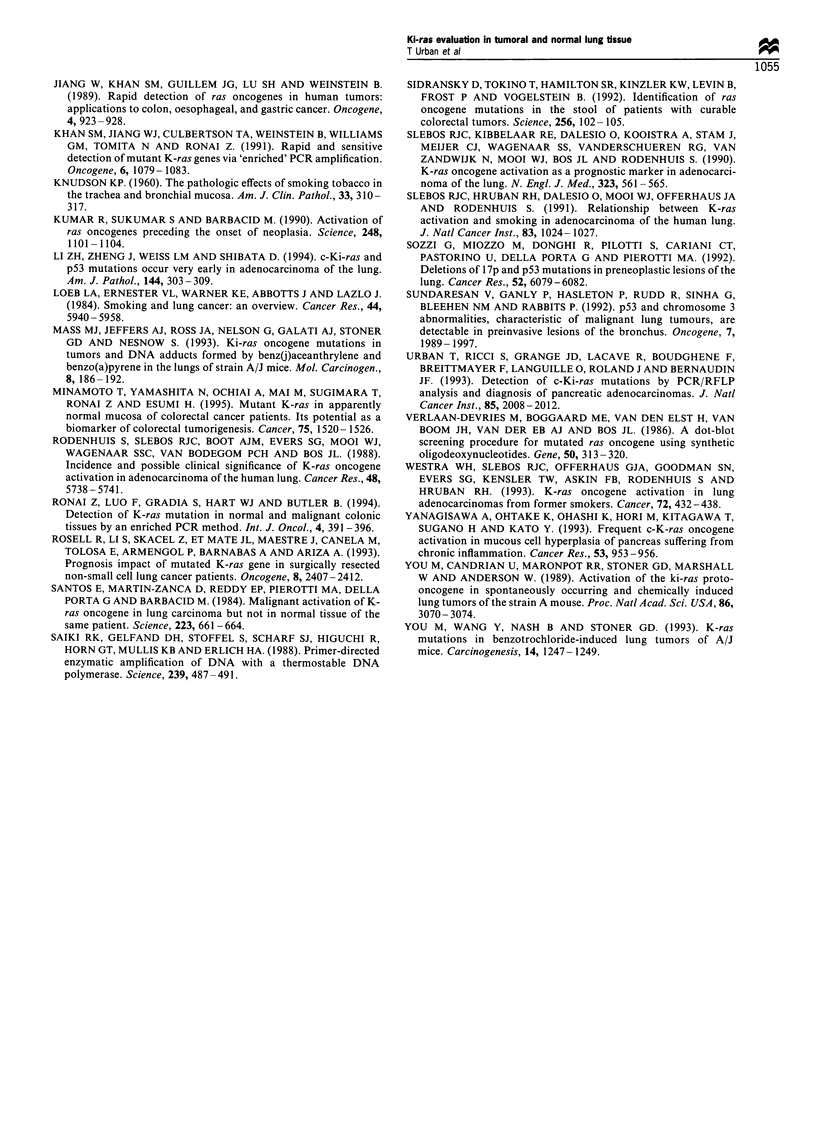# Codon 12 Ki-ras mutation in non-small-cell lung cancer: comparative evaluation in tumoural and non-tumoural lung.

**DOI:** 10.1038/bjc.1996.488

**Published:** 1996-10

**Authors:** T. Urban, S. Ricci, R. Lacave, M. Antoine, M. Kambouchner, F. Capron, J. F. Bernaudin

**Affiliations:** Division of Pneumology, Hôpital Saint-Antoine, Paris, France.

## Abstract

**Images:**


British Journal of Cancer (1996) 74, 1051-1055
© 1996 Stockton Press All rights reserved 0007-0920/96 $12.00

### Codon 12 Ki-*ras* mutation in non-small-cell lung cancer: comparative evaluation in tumoural and non-tumoural lung

T Urban[1], S Ricci[2], R Lacave[2], M Antoine[3], M Kambouchner[4], F Capron[5] and J-F Bernaudin[2]

*[1]Division of Pneumology, Hôpital Saint-Antoine, 184 rue du Faubourg Saint-Antoine, 75012 Paris, France; [2]Laboratory of Histology and Tumor Biology and [3]Laboratory of Pathology, Hôpital Tenon, 4 rue de la Chine, 75020 Paris, France; [4]Laboratory of Pathology, Hôpital Avicenne, 125 rue de Stalingrad, 95000 Bobigny, France; [5]Laboratory of Pathology, Hôpital Béclère, 157 rue de la porte de Trivaux, 92140 Clamart, France.*

**Summary** Ki-*ras* activation by point mutation on codon 12 has been reported in non-small-cell lung carcinomas and in various models of experimental lung tumours induced by chemical carcinogens. The cellular targets for carcinogenic compounds of tobacco smoke are usually considered to be the cells of the bronchial mucosa or alveolar epithelium. However, little is known about preneoplastic events in bronchopulmonary carcinogenesis. The hypothesis of the presence of widespread target cells containing Ki-*ras* mutation was investigated by evaluating concurrent neoplastic and non-neoplastic bronchial and alveolar samples from 51 patients with non-small-cell lung carcinomas. The polymerase chain reaction–restriction fragment length polymorphism (PCR–RFLP) method used can detect one cell with a mutation on codon 12 among $10^2$ normal cells. In tumour samples, a mutation was detected in 20% of adenocarcinomas, but in none of the adenosquamous or squamous cell carcinomas. No mutation was detected in the non-neoplastic bronchial or parenchymal samples. When using an enriched PCR–RFLP method detecting one mutated allele among $10^3$ normal alleles a mutation was detected in 23% of adenocarcinomas. In conclusion, Ki-*ras* activation by mutation on codon 12 was not observed in non-neoplastic bronchial or parenchymal tissues in patients with bronchopulmonary cancers and does not appear to be a genetic event present in non-malignant epithelial target cells exposed to tobacco smoke.

**Keywords:** lung carcinoma; ki-*ras*; oncogene

The Ki-*ras* proto-oncogene has been shown to be activated by point mutations in a wide variety of human and experimental carcinomas (Barbacid, 1987; Bos, 1989). Dutch researchers reported that in lung carcinomas Ki-*ras* mutations were found exclusively in adenocarcinomas with a frequency of 30% in smokers and 5% in non-smokers (Bos, 1989; Slebos *et al.*, 1991). In neoplastic tissues more than 90% of Ki-*ras* mutations occurred in codon 12 (Bos, 1989; Slebos *et al.*, 1991). In contrast, in another series from Spain, Ki-*ras* mutations were not only detected in adenocarcinomas, but the majority of Ki-*ras* mutations were present in squamous cell carcinomas (Rosell *et al.*, 1993). In experimental models of lung tumours conducted in different mouse strains, various chemical carcinogens have been demonstrated to induce tumours harbouring a Ki-*ras* mutation restricted to codon 12 [You *et al.*, 1989, 1993; Mass *et al.*, 1993). These human and experimental data suggest that codon 12 of Ki-*ras* may be a specific target for the mutagenic activity of various compounds of tobacco smoke (Husafvel-Pursiaisen *et al.*, 1993).

In colonic carcinogenesis, Ki-*ras* mutation has been shown to be a preneoplastic event (Burmer and Loeb, 1989). In contrast, little is known about preneoplastic events in bronchopulmonary carcinogenesis. However, recent reports demonstrated that Ki-*ras* and p53 mutations occur very early in the development of pulmonary adenocarcinomas (Sozzi *et al.*, 1992; Sundaresan *et al.*, 1992; Benett, 1993; Li *et al.*, 1994). The cellular targets for the carcinogenic compounds of tobacco smoke are usually considered to be either the bronchial mucosa or alveolar epithelium (Carney, 1991). Therefore, the hypothesis of the presence of widespread target cells containing Ki-*ras* mutations in the respiratory tract as already shown for the suppressor gene p53 has to be considered (Sozzi *et al.*, 1992; Sundaresan *et al.*, 1992; Benett 1993; Li *et al.*, 1994).

Correspondence: JF Bernaudin, Laboratoire d'Histologie-Biologie Tumorale, Pavillon Proust–Hôpital Tenon, 4 rue de la Chine, 75020 Paris, France
Received 7 July 1995; revised 27 March 1996; accepted 17 April 1996

Consequently, the present study was designed to investigate the presence of activated Ki-*ras* by mutation in codon 12 not only in adenocarcinomas, but also in various non-small-cell bronchopulmonary cancer tissues and in neighbouring and distant non-neoplastic bronchial and lung tissues.

### Materials and Methods

### *Tissue specimens*

Tissue specimens were collected in 68 smokers or ex-smokers who were patients for thoracotomy undertaken with curative intent. The resected material was transported to the pathology department, and after examination by the pathologist, a representative part of the tumour was snap-frozen and stored at −70°C until analysis. To increase the sensitivity of detection, macroscopically neoplastic tissue was collected under a dissection microscope.

Concomitantly, in 51 patients, non-neoplastic bronchial and parenchymal specimens were also collected close to and away from the tumour and snap-frozen at −70°C. Tissue samples were also embedded in paraffin wax for histological analysis and classified according to the WHO classification into squamous cell carcinomas (SCC), adenocarcinomas (AC) or adenosquamous carcinomas (ASC).

### *DNA evaluation by polymerase chain reaction (PCR) amplification and restriction fragment length polymorphism (RFLP) analysis*

A rapid method of extraction of DNA from tissues and cell cultures was performed according to Higuchi (1989). Ki-*ras* codon 12 sequences were amplified according to Jiang *et al.* (1989) using a 5′-end primer that contains a C substitution at the first position of codon 11 creating a *Bst*NI site which overlaps the first two nucleotides of codon 12. The 3′ primer also contains a substitution creating a control *Bst*NI site.

The primers, synthesized by a DNA synthesiser (Applied Biosystems) and purified by high performance liquid chromatography (HPLC) (Genset, Paris), were Ki-*ras* 5′: 5′-ACT GAA TAT AAA CTT GTG GTA GTT GGA CCT-3′ and Ki-*ras* 3′: 5-TCA AAG AAT GGT CCT GGA CC-3′. DNA was amplified according to Saiki *et al.* (1988) using a Perkin-Elmer Cetus thermal cycler. DNA was denatured at 94°C for 10 min and subsequently amplified for 40 cycles. The PCR reaction was carried out in 0.1 ml containing 50 mM potassium chloride, 10 mM Tris-HCl, pH 8.3, 1.5 mM magnesium chloride, 2.0 mM of each dNTP, with 350 ng of each primer and 2.5 units of *Taq* DNA polymerase (Beckman). The reaction mixture was overlayed with mineral oil. Each cycle included a 1 min denaturation step at 94°C, a 1 min annealing step at 55°C and a 1.5 min elongation step at 72°C. PCR reaction product (10 μl) was analysed by 2% agarose gel electrophoresis. Specimens with negative results were re-evaluated in a second separate PCR amplification. Aliquots (20 μl) of the PCR reaction product were then digested with the restriction enzyme *Bst,/it*>NI (Boehringer Mannheim, France) at 37°C for 2 h and electrophoresed through an 8% polyacrylamide gel. The results were analysed after ethidium bromide staining and UV transillumination.

*Codon 12 mutation identification by specific oligonucleotide hybridisation*

Ten samples (six wild-type and four mutated codon 12 of c-K-*ras* genes) were studied by specific oligonucleotide hybridisation. After extraction, the DNA was heat denatured and used for *in vitro* amplification. The PCR procedure, oligonucleotide sequences, hybridisation conditions in ammonium tetramethylchloride and composition of the seven probes used have been described previously by Verlaan de Vries *et al.* (1986).

*Enriched PCR/RFLP analysis*

An enriched PCR/RFLP method derived from Kahn *et al.* (1991) was concurrently performed in non-tumoral and tumoral samples from lungs of 32 patients with adenocarcinomas. Ki-*ras* codon 12 sequences were amplified in two steps. Primers used were K-*ras* 5′ (as described above), K-*ras* 3′ wild type (5′-TCA AAG AAT GGT CCT GCA CC-3′) and K-*ras* 3′ (as described above).

*First step amplification* The PCR reaction was carried out in 0.1 ml containing 50 mM potassium chloride, 10 mM Tris-HCl, pH 8.3, 1.5 mM magnesium chloride, 0.2 mM dNTPs and 2.5 units of *Taq* DNA polymerase (Beckman). Primer concentrations were 10 ng each of K-*ras* 5′ and Ki-*ras* 3′ wild-type. The reaction mixture was overlayed with mineral oil. Each cycle included a 1 min denaturation step at 94°C, a 1 min 30 s annealing step at 56°C and a 2 min elongation step at 72°C, for a total of 15 cycles.

*Intermediate digestion* Aliquots (5 μl) of the first PCR reaction were digested with 20 units of the restriction enzyme *Bst*NI (Boehringer Mannheim, France) in a final volume of 10 μl at 37°C for 3 h under conditions recommended by the supplier.

*Second step amplification* Digested mixture (10 μl) was used in the second amplification step. These aliquots were diluted to a final volume of 50 μl as described above. Primer concentrations were 100 ng each of Ki-*ras* 5′ and Ki-*ras* 3′, and amplification was performed for 30 cycles as above.

*RFLP analysis* Aliquots (20 μl) of the products obtained after the second step were digested with the restriction enzyme *Bst*NI (10 units) at 37°C for 2 h in a final volume of 30 μl. The results were analysed after ethidium bromide staining and UV transillumination.

*Control cell lines*

SW480 (homozygous for a mutated Ki-*ras* gene codon 12, valine, GTT) and HT29 (homozygous for the wild-type Ki-*ras* gene codon 12, glycine, GGT) human colon carcinoma cell lines were obtained from the American Type Culture Collection (ATCC, Rockville, MD, USA). All were grown in the prescribed media.

### Results

*One-step PCR amplification and RFLP analysis*

Amplification of Ki-*ras* codon 12 sequence gave a 157 bp fragment. Digestion of wild-type codon 12 sequence with *Bst* NI generated a 114 bp fragment, while, when a mutation was present in codon 12, *Bst*NI digestion generated a 143 bp fragment, as shown in Figure 1. Therefore, the presence of a 143 bp fragment after digestion is the hallmark of the presence of mutated Ki-*ras* genes on codon 12.

*Detection of codon 12 c-Ki-*ras *mutation in SW480 and HT29 cell lines*

Two human cell lines with documented codon 12 Ki-*ras* gene were examined to validate the method. SW480 is derived from a human colon carcinoma and is a homozygous mutant (GGT to GTT) (Capon *et al.*, 1983). HT29 is also derived from a human colon carcinoma and has homozygous wild-type alleles (codon 12 Ki-*ras*, GGT). PCR amplification and *Bst*NI RFLP analysis of DNA from the SW 480 cell line showed only a 143 bp fragment confirming the homozygous mutation. Analysis of DNA from the HT29 cell line showed a wild-type 114 bp fragment. Analysis of a mixture of DNA from the two cell lines showed two fragments of 143 and 114 bp respectively. The sensitivity of the assay was therefore tested by a series of titration experiments. The threshold of detection was one cell with a homozygous mutation in the midst of $10^2$ cells with a wild-type Ki-*ras* gene (Figure 2) (Urban *et al.*, 1993).

**Figure 1** Photograph of DNA electrophoresis through an 8% polyacrylamide gel after ethidium bromide staining and UV analysis. Left lane molecular weight markers. The basepair number is indicated in the right margin. Lanes 1–2, *Bst*NI digestion of PCR product after amplification of DNA from SW480 cell line with homozygous mutation showing a single 143 bp fragment. Lanes 3–4, *Bst*NI digestion of PCR product after amplification of DNA from wild-type HT29 cell line showing a single 114 bp fragment. Lanes 5 and 6, *Bst*NI digestion of PCR product after amplification of DNA from samples with heterozygous mutation, showing 114 and 143 bp fragments. Lane 7, Non-digested PCR product showing a 157 bp fragment.

**Figure 2** Sensitivity of one-step PCR amplification and RFLP analysis. Photograph of DNA electrophoresis through an 8% polyacrylamide gel after ethidium bromide staining and UV analysis. Left and right lanes, molecular weight markers. The basepair number is indicated in the right margin. Lane 1, *Bst*NI digestion of PCR product after amplification of DNA from wild-type HT29 cell line showing a single 114 bp fragment. Lanes 2–6, *Bst*NI digestion of PCR product after amplification of a mixture of DNA from HT29 and SW480 cell lines, with the following SW480/HT29 DNA ratio: lane 2, 1/10000; lane 3, 1/1000; lane 4, 1/100; lane 5, 1/10; lane 6, 1/1. The 114 bp fragment is generated from HT29-Ki-*ras* gene. In contrast the 143 bp fragments indicated the presence of a SW480 Ki-*ras* gene mutated on codon 12. The sensitivity ratio was 1/100. Lane 7, *Bst*NI digestion of PCR product after amplification of DNA from SW480 cell line with homozygous mutation showing a single 143 bp fragment.

**Figure 3** Sensitivity of 'enriched' PCR amplification and RFLP analysis. Photograph of DNA electrophoresis through an 8% polyacrylamide gel after ethidium bromide staining and UV analysis. Left and right lanes, molecular weight markers. The basepair number is indicated in the right margin. Lane 1, *Bst*NI digestion of PCR product after amplification of DNA from HT29 cell line showing a single 114 bp fragment. Lanes 2–3, *Bst*NI digestion of PCR product after amplification of a mixture of DNA from HT29 and SW480 cell lines, with the following SW480/HT29 DNA ratio: lane 2, 1/100 000; lane 3, 1/10 000. The 114 bp fragment is generated from HT29-Ki-*ras* gene. The 143 bp fragment is not detected. Lane 4–7, *Bst*NI digestion of PCR product after amplification of a mixture of DNA from HT29 and SW480 cell lines, with the following SW480/HT29 DNA ratio: lane 4, 1/1000; lane 5, 1/100, lane 6, 1/10; lane 7, 1/1. The 114 bp fragment is generated from HT29-Ki-*ras* gene. In contrast the 143 bp fragments indicated the presence of a SW480 Ki-*ras* gene mutated on codon 12. The sensitivity ratio is 1/1000. Lane 8 *Bst*Ni digestion of PCR product after amplification of DNA from SW480 cell line showing a single 143 bp fragment.

When using the enriched PCR–RFLP method (Kahn *et al.*, 1991), the threshold of detection was one cell with homozygous mutation in the midst of $10^3$ cells with a wild-type Ki-*ras* gene (Figure 3).

*Detection of codon 12 c-Ki-*ras *mutation in patient samples*

The results of the DNA evaluation by PCR amplification and RFLP analysis (PCR–RFLP method) are shown in Tables I and II.

When using the one step PCR–RFLP method no mutation involving the codon 12 of Ki-*ras* gene was detected in the 20 squamous cell and four adenosquamous cell carcinomas. In contrast a mutation was detected in 9/44 (20.5%) of the adenocarcinomas. When using the enriched PCR–RFLP method, a mutation was detected in 10/44 (23%) of the adenocarcinomas.

As a control of the method, ten samples were studied by specific oligonucleotide hybridisation. No mutation was detected in the six samples found to be of the wild type by PCR–RFLP analysis. In contrast, the four samples in which a mutated codon 12 of Ki-*ras* was detected by PCR–RFLP analysis, the mutation could be specific (2:GGT→GTT; 1:GGT→GCT;1:GGT→TGT).

No mutation of the codon 12 of Ki-*ras* gene was detected in the non-neoplastic bronchial and parenchymal specimens even in the samples from lungs with adenocarcinomas harbouring a Ki-*ras* mutation (Table II).

**Table I** Detection of a codon 12 Ki-*ras* mutation by one-step PCR–RLFP method in 68 malignant and 51 non-neoplastic tissues from patients with non-small-cell lung carcinoma

| | *Malignant tissue* | | *Non-neoplastic tissue* | |
|---|---|---|---|---|
| | n | *Mutated Ki*-ras | n | *Mutated Ki*-ras |
| Adenocarcinomas | 44 | 9 | 32 | 0 |
| Squamous cell carcinomas | 20 | 0 | 15 | 0 |
| Adenosquamous cell carcinomas | 4 | 0 | 4 | 0 |

**Table II** Presence of ki-*ras* mutations in lung adenocarcinomas and apparently normal tissues evaluated by one-step PCR–RFLP and enriched PCR–RFLP methods

| | | *Mutated Ki*-ras | |
|---|---|---|---|
| | n | *One-step PCR* | *Enriched PCR* |
| Adenocarcinomas | 44 | 9 | 10 |
| Concurrent samples | 32 | | |
| Malignant tissue | | 4 | 5 |
| Non-neoplastic tissue[a] | | 0 | 0 |

[a] Apparently normal bronchial and lung tissue.

### Discussion

The methods chosen for the detection of a mutation in codon 12 of the Ki-*ras* gene were a combination of PCR amplification of DNA and RFLP analysis (Jiang *et al.*, 1989). These methods offer several advantages. They are highly specific, as the loss of the restriction site at the target is diagnostic for the presence of a mutation. An additional restriction site offers a control of the restriction enzyme digestion. In the present study the one-step PCR–RFLP analysis was shown to be able to detect a K-*ras* mutation when it is present in 1% of cells studied. When using the enriched PCR–RFLP procedure, one mutated allele could be detected among $10^3$ normal alleles.

In the present series of 68 non-small-cell lung carcinomas, activation of Ki-*ras* by a mutation on codon 12 was observed in ten of the 44 adenocarcinomas. Such a frequency (23%) is in agreement with most of the previous reports (Rodenhuis *et al.*, 1988; Slebos *et al.*, 1990). No mutation was detected in the 20 squamous and four adenosquamous cell carcinomas investigated. This result is in line with most of the reports which demonstrated that Ki-*ras* mutations were restricted to adenocarcinomas except for Rosell *et al.* (1993), who observed Ki-*ras* mutations in squamous cell carcinomas. In this last series, mutations were also detected in codon 61 which was not investigated in the present study, focusing on codon 12 of Ki-*ras*.

In experimental models of lung tumours induced in various murine strains, the presence of Ki-*ras* mutation is a particularly reproducible event. For example, in the strain A mouse highly susceptible to spontaneous and inducible lung tumours, Ki-*ras* point mutations in spontaneous lung tumours were found in both codons 12 (60%) and 61 (30%) (You *et al.*, 1989, 1993; Mass *et al.*, 1993). After tumour induction in this mouse strain, the pattern of Ki-*ras* mutation was different according to the carcinogen used: in codon 12 for 100% of tumours after induction by methyl nitrosourea and 93% after benzo[*a*]pyrene exposure, in contrast, 90% of the mutations after ethyl carbonate exposure were situated in codon 61 (You *et al.*, 1989). Benzo[*a*]pyrene is considered to be a major carcinogenic compound of tobacco smoke (Loeb *et al.*, 1984). In human lung and pancreatic adenocarcinomas, the presence of codon 12 Ki-*ras* mutation has been shown to be related to tobacco smoke exposure (Hruban *et al.*, 1993; Westra *et al.*, 1993).

Ras activation by mutation, particularly Ki-*ras*, has been shown to be present in a wide variety of human neoplasms (Bos, 1989). Moreover, it has been suggested that Ki-*ras* oncogene activation could precede the onset of neoplasia (Kumar *et al.*, 1990). For colonic carcinoma, Ki-*ras* activation by mutation was observed in adenomas before the development of non-invasive cancer and is therefore under investigation for the survey of at-risk patients (Sidransky *et al.*, 1992). Moreover, when using an enriched PCR method, Ki-*ras* mutations could be detected in normal colonic mucosa at a distance from the tumour (Ronai *et al.*, 1994; Minamoto *et al.*, 1995). For pancreatic carcinomas, Ki-*ras* mutations have been detected in mucous hyperplasia associated with pancreatitis, suggesting they may be precancerous epithelial modifications (Yanagisawa *et al.*, 1993). For lung adenocarcinomas, c-*Ki-ras* mutations occur early since they have been detected in all stages of lung cancer (Li *et al.*, 1994). Moreover, these mutations are considered to be an irreversible event (Westra *et al.*, 1993).

Widespread dysplastic lesions have been observed in bronchi of cigarette smokers increasing in a dose-dependent manner according to the number of cigarettes smoked (Auerbach *et al.*, 1979). Multiple mutations are necessary for the transformation of epithelial cells and the exact order in which they are acquired is difficult to ascertain.

p53 mutations present in both squamous cell carcinomas and adenocarcinomas have been detected in association with lesions considered to be precancerous such as bronchial squamous metaplasia or dysplastic tissue (Sozzi *et al.* 1992; Sundaresan *et al.*, 1992; Benett, 1993; Li *et al.*, 1994). Similar results were reported for the loss of 3p allele observed in most bronchopulmonary carcinomas (Brauch *et al.*, 1987).

In contrast, in our series, as for the patient investigated by Santos *et al.* (1984), no Ki-*ras* mutations in codon 12 were detected in bronchial and lung parenchymal samples from patients with non-small-cell lung carcinomas, even when mutations were present in tumour samples. Moreover, no Ki-*ras* mutation was detected in dysplastic bronchial epithelium associated with lung adenocarcinomas (Li *et al.*, 1994). However, rare cells with mutations below the threshold of detection may be present.

Clements *et al.* (1995) recently reported Ki-*ras* mutation in non-malignant bronchial tissue in patients with lung carcinomas. Such a discrepancy can be explained by the fact that Clements *et al.* evaluated samples from the carina of proximal bronchi. In contrast, we deliberately studied peripheral bronchial or alveolar lung tissue as they are suspected to be possible cellular targets for carcinogens in adenocarcinomas. Bronchial carina has been shown to be the site of epithelial changes in response to constant exposure to airborne contaminants (Knudtson *et al.*, 1960). However, it is not suspected to be the site of occurrence of adenocarcinomas.

In conclusion, Ki-*ras* mutations were detected in proximal bronchial carina in another study. In the present study, Ki-*ras* mutations were not detected in peripheral bronchial and lung parenchyma associated with lung tumours, particularly adenocarcinomas. These results suggest that these mutations do no occur in widespread cellular genomes induced by tobacco smoke in peripheral bronchial and lung tissue.

**Acknowledgements**

This study was supported by grants from Recherche Clinique, Assistance Publique-Hôpitaux de Paris, Ligue Contre le Cancer and Association ACTT. I Höpfel and V Gerber are gratefully acknowledged for secretarial assistance.

### References

AUERBACH O, HAMMOND EC AND GARFINKEL L. (1979). Changes in bronchial epithelium in relation to cigarette smoking, 1955–1960 *vs* 1970–1977. *N. Engl. J. Med.*, **300**, 381–386.

BARBACID M. (1987). *ras* genes. *Annu. Rev. Biochem.*, **56**, 779–827.

BENETT WP, COLBY TV, TRAVIS WD, BORKOWSKI A, JONES RT, LANE DP, METCALF RA, SAMET JM, TAKESHIMA Y, GU JR, VÄHÄKANGAS KH, SOINI Y, PÄÄKKOÖ P, WELSH JA, TRUMP BF AND HARRIS CC. (1993). p53 protein accumulates frequently in early bronchial neoplasia. *Cancer Res.*, **53**, 4817–4822.

BOS JL. (1989). *ras* oncogenes in human cancer: a review. *Cancer Res.*, **49**, 4682–4689.

BRAUCH H, JONHSON B, HOVIS J, YANO T, GAZDAR A, PETTENGILL O, GRAZIANO S, SORENSON G, POIESZ B, MINNA J, LINEHAN M AND ZBAR B. (1987). Molecular analysis of the short arm of chromosome 3 in small cell and non-small cell lung carcinoma of the lung. *N. Engl. J. Med.*, **317**, 109–113.

BURMER GC AND LOEB L. (1989). Mutations in the K-*ras* oncogene during progressive stages of human colon carcinoma. *Proc. Natl Acad. Sci. USA*, **86**, 2403–2407.

CAPON DJ, SEEBURG PH, MC GRATH J, HAYFLICK JS, EDMAN U, LEVINSON AD AND GOEDEL DV. (1983). Activation of Ki-*ras* gene in human colon and lung carcinomas by two different point mutations. *Nature*, **304**, 507–513.

CARNEY DN. (1991). Lung cancer biology. *Curr. Opin. Cancer*, **3**, 288–296.

CLEMENTS NC, NELSON MA. WYMER JA, SAVAGE C, AGUIRRE M AND GAREWAL H. (1995). Analysis of K-*ras* gene mutations in malignant and non-malignant endobronchial tissue obtained by fibreoptic bronchoscopy. *Am. J. Respir. Crit. Care Med.*, **152**, 1374–1378.

HIGUCHI R. (1989). Simple and rapid preparation of samples for PCR. In *PCR Technology. Principles and Applications for DNA Amplification*. Erlich HA (ed.) pp. 35–36. Stockton Press: New York.

HRUBAN RH, VAN MANSFELD ADM, OFFERHAUS GJA, VAN WEERING DH, ALISON DC, GOODMAN SN, KENSLER TW, BOSE KK, CAMERON JL AND BOS JL. (1993). K-*ras* oncogene activation in adenocarcinoma of the human pancreas. *Am. J. Pathol.*, **143**, 545–554.

HUSAFVEL-PURSIAISEN K, HACKMAN P, RIDANPÄÄ M, ANTTILAS S, KARJALAINEN A, TARTANEN T, TAIKINA-AHO O, HEIKKILA L AND VAINIO H. (1993). K-*ras* mutations in human adenocarcinoma of the lung with smoking and occupational exposure to asbestosis. *Int. J. Cancer*, **53**, 250–256.

JIANG W, KHAN SM, GUILLEM JG, LU SH AND WEINSTEIN B. (1989). Rapid detection of *ras* oncogenes in human tumors: applications to colon, oesophageal, and gastric cancer. *Oncogene*, **4**, 923-928.

KHAN SM, JIANG WJ, CULBERTSON TA, WEINSTEIN B, WILLIAMS GM, TOMITA N AND RONAI Z. (1991). Rapid and sensitive detection of mutant K-*ras* genes via 'enriched' PCR amplification. *Oncogene*, **6**, 1079-1083.

KNUDSON KP. (1960). The pathologic effects of smoking tobacco in the trachea and bronchial mucosa. *Am. J. Clin. Pathol.*, **33**, 310-317.

KUMAR R, SUKUMAR S AND BARBACID M. (1990). Activation of *ras* oncogenes preceding the onset of neoplasia. *Science*, **248**, 1101-1104.

LI ZH, ZHENG J, WEISS LM AND SHIBATA D. (1994). c-Ki-*ras* and p53 mutations occur very early in adenocarcinoma of the lung. *Am. J. Pathol.*, **144**, 303-309.

LOEB LA, ERNESTER VL, WARNER KE, ABBOTTS J AND LAZLO J. (1984). Smoking and lung cancer: an overview. *Cancer Res.*, **44**, 5940-5958.

MASS MJ, JEFFERS AJ, ROSS JA, NELSON G, GALATI AJ, STONER GD AND NESNOW S. (1993). Ki-*ras* oncogene mutations in tumors and DNA adducts formed by benz(j)aceanthrylene and benzo(a)pyrene in the lungs of strain A/J mice. *Mol. Carcinogen.*, **8**, 186-192.

MINAMOTO T, YAMASHITA N, OCHIAI A, MAI M, SUGIMARA T, RONAI Z AND ESUMI H. (1995). Mutant K-*ras* in apparently normal mucosa of colorectal cancer patients. Its potential as a biomarker of colorectal tumorigenesis. *Cancer*, **75**, 1520-1526.

RODENHUIS S, SLEBOS RJC, BOOT AJM, EVERS SG, MOOI WJ, WAGENAAR SSC, VAN BODEGOM PCH AND BOS JL. (1988). Incidence and possible clinical significance of K-*ras* oncogene activation in adenocarcinoma of the human lung. *Cancer Res.*, **48**, 5738-5741.

RONAI Z, LUO F, GRADIA S, HART WJ AND BUTLER B. (1994). Detection of K-*ras* mutation in normal and malignant colonic tissues by an enriched PCR method. *Int. J. Oncol.*, **4**, 391-396.

ROSELL R, LI S, SKACEL Z, ET MATE JL, MAESTRE J, CANELA M, TOLOSA E, ARMENGOL P, BARNABAS A AND ARIZA A. (1993). Prognosis impact of mutated K-*ras* gene in surgically resected non-small cell lung cancer patients. *Oncogene*, **8**, 2407-2412.

SANTOS E, MARTIN-ZANCA D, REDDY EP, PIEROTTI MA, DELLA PORTA G AND BARBACID M. (1984). Malignant activation of K-*ras* oncogene in lung carcinoma but not in normal tissue of the same patient. *Science*, **223**, 661-664.

SAIKI RK, GELFAND DH, STOFFEL S, SCHARF SJ, HIGUCHI R, HORN GT, MULLIS KB AND ERLICH HA. (1988). Primer-directed enzymatic amplification of DNA with a thermostable DNA polymerase. *Science*, **239**, 487-491.

SIDRANSKY D, TOKINO T, HAMILTON SR, KINZLER KW, LEVIN B, FROST P AND VOGELSTEIN B. (1992). Identification of *ras* oncogene mutations in the stool of patients with curable colorectal tumors. *Science*, **256**, 102-105.

SLEBOS RJC, KIBBELAAR RE, DALESIO O, KOOISTRA A, STAM J, MEIJER CJ, WAGENAAR SS, VANDERSCHUEREN RG, VAN ZANDWIJK N, MOOI WJ, BOS JL AND RODENHUIS S. (1990). K-*ras* oncogene activation as a prognostic marker in adenocarcinoma of the lung. *N. Engl. J. Med.*, **323**, 561-565.

SLEBOS RJC, HRUBAN RH, DALESIO O, MOOI WJ, OFFERHAUS JA AND RODENHUIS S. (1991). Relationship between K-*ras* activation and smoking in adenocarcinoma of the human lung. *J. Natl Cancer Inst.*, **83**, 1024-1027.

SOZZI G, MIOZZO M, DONGHI R, PILOTTI S, CARIANI CT, PASTORINO U, DELLA PORTA G AND PIEROTTI MA. (1992). Deletions of 17p and p53 mutations in preneoplastic lesions of the lung. *Cancer Res.*, **52**, 6079-6082.

SUNDARESAN V, GANLY P, HASLETON P, RUDD R, SINHA G, BLEEHEN NM AND RABBITS P. (1992). p53 and chromosome 3 abnormalities, characteristic of malignant lung tumours, are detectable in preinvasive lesions of the bronchus. *Oncogene*, **7**, 1989-1997.

URBAN T, RICCI S, GRANGE JD, LACAVE R, BOUDGHENE F, BREITTMAYER F, LANGUILLE O, ROLAND J AND BERNAUDIN JF. (1993). Detection of c-Ki-*ras* mutations by PCR/RFLP analysis and diagnosis of pancreatic adenocarcinomas. *J. Natl Cancer Inst.*, **85**, 2008-2012.

VERLAAN-DEVRIES M, BOGGAARD ME, VAN DEN ELST H, VAN BOOM JH, VAN DER EB AJ AND BOS JL. (1986). A dot-blot screening procedure for mutated *ras* oncogene using synthetic oligodeoxynucleotides. *Gene*, **50**, 313-320.

WESTRA WH, SLEBOS RJC, OFFERHAUS GJA, GOODMAN SN, EVERS SG, KENSLER TW, ASKIN FB, RODENHUIS S AND HRUBAN RH. (1993). K-*ras* oncogene activation in lung adenocarcinomas from former smokers. *Cancer*, **72**, 432-438.

YANAGISAWA A, OHTAKE K, OHASHI K, HORI M, KITAGAWA T, SUGANO H AND KATO Y. (1993). Frequent c-K-*ras* oncogene activation in mucous cell hyperplasia of pancreas suffering from chronic inflammation. *Cancer Res.*, **53**, 953-956.

YOU M, CANDRIAN U, MARONPOT RR, STONER GD, MARSHALL W AND ANDERSON W. (1989). Activation of the ki-*ras* proto-oncogene in spontaneously occurring and chemically induced lung tumors of the strain A mouse. *Proc. Natl Acad. Sci. USA*, **86**, 3070-3074.

YOU M, WANG Y, NASH B AND STONER GD. (1993). K-*ras* mutations in benzotrochloride-induced lung tumors of A/J mice. *Carcinogenesis*, **14**, 1247-1249.